# High-sensitivity cardiac troponin T as an independent predictor of stroke in patients admitted to an emergency department with atrial fibrillation

**DOI:** 10.1371/journal.pone.0212278

**Published:** 2019-02-12

**Authors:** Mehrshad Vafaie, Evangelos Giannitsis, Matthias Mueller-Hennessen, Moritz Biener, Elena Makarenko, Buelent Yueksel, Hugo A. Katus, Kiril M. Stoyanov

**Affiliations:** Department of Cardiology, Angiology and Pulmonology, Heidelberg University Hospital, Heidelberg, Germany; Klinikum Region Hannover GmbH, GERMANY

## Abstract

**Aims:**

Elevated levels of high-sensitivity cardiac troponin T (hsTnT) are associated with adverse outcomes in numerous patient populations. Their value in prediction of stroke risk in patients with atrial fibrillation (AF) is in debate.

**Methods:**

The study population included 2898 consecutive patients presenting with AF to the emergency department of the Department of Cardiology, Heidelberg University Hospital. Associations between hsTnT and stroke risk were assessed using multivariable Cox regression.

**Results:**

Elevated hsTnT levels (>14 ng/L) were associated with increased risk of stroke. Even after adjustment for various risk factors, elevated hsTnT remained independently associated with stroke risk in patients with AF, adjusted hazard ratio 2.35 [95% confidence interval (CI): 1.26–4.36] (P = 0.007). These results were consistent across important subgroups (age, renal function, ejection fraction, CHA_2_DS_2_-VASc score and main admission diagnosis). For hsTnT, area under the receiver-operating-characteristic curve (AUC) was 0.659 [95% CI: 0.575–0.742], compared to 0.610 [95% CI: 0.526–0.694] for the CHA_2_DS_2_-VASc score. Inclusion of hsTnT in the multivariable model for stroke risk prediction consisting of all variables of the CHA_2_DS_2_-VASc score was associated with a significant improvement of its discriminatory power.

**Conclusion:**

Elevated hsTnT levels are significantly associated with higher risk of stroke and provide prognostic information independent of CHA_2_DS_2_-VASc score variables. Measurement of hsTnT may improve prediction of stroke risk in patients presenting to an emergency department with AF as compared to risk stratification based only on clinical variables.

## Introduction

Atrial fibrillation (AF) is the most common type of sustained arrhythmia. Patients with certain risk factors have a significantly increased risk of stroke [1]. Prevalence of AF increases with age and reaches almost 9% in patients aged 80–89 [2]. It is important to note that cardiac thromboembolism accounts for 15 to 20% of all strokes [3].

The risk of thromboembolism in patients with AF is commonly assessed using clinical variables such as congestive heart failure (CHF), hypertension, age, diabetes mellitus, and prior stroke or transient ischemic attack (TIA) in the initial CHADS_2_ risk score, as well as in the currently widely recommended CHA_2_DS_2_-VASc risk score, which includes additional variables (vascular disease and sex) [4,5]. However, the performance of this score is limited when measured by C statistic of 0.606 [95% confidence interval (CI): 0.513–0.699], which is only slightly better than the CHADS_2_-Score (0.561 [95% CI: 0.450–0.672]) [5].

Current European Society of Cardiology guidelines for the management of AF recommend anticoagulation when CHA_2_DS_2_-VASc score is 2 or higher in men and 3 or higher in women, and refrain from anticoagulation in men with a score of 0 and women with a score of 1 only due to female gender. With a CHA_2_DS_2_-VASc score of 1 in men and 2 in women, consideration of anticoagulation is recommended depending on expected stroke rate, bleeding risk and patient preference [4].

Cardiac troponin elevations are associated with underlying heart disease and adverse events in patients with acute coronary syndrome (ACS) [6], stable coronary artery disease (CAD) [7], chronic heart failure [8], and in community-based populations [9]. It has been demonstrated that elevated levels of cardiac troponin are highly prevalent among patients with acute ischemic stroke and are associated with higher mortality [10,11]. Suggested pathophysiologic mechanisms include neurally mediated autonomic dysregulation and cardiac injury secondary to sympathoadrenal activation [12].

The prognostic value of biomarkers in patients with AF regarding stroke risk prediction has not been established conclusively. Current evidence for association of elevated cardiac troponin levels with higher stroke risk in patients with AF is based on secondary analyses of randomized controlled trials (RCT)–such as the Randomized Evaluation of Long-Term Anticoagulation Therapy (RE-LY) trial [13] and the Apixaban for the Prevention of Stroke in Subjects With Atrial Fibrillation (ARISTOTLE) trial [14]. Recently, novel risk scores for patients with AF using biomarkers have been derived from RCT populations [15–17]. In the setting of an emergency department, we have shown, that elevated cardiac troponin levels are associated with higher mortality in patients with AF [18].

We aimed to assess real-world data by investigating the association of high-sensitivity cardiac troponin T (hsTnT) levels with stroke risk in an unselected population of patients presenting to an emergency department with AF, especially in relation to clinical risk factors and the CHA_2_DS_2_-VASc risk score.

## Methods

### Study population and design

**Fig 1:**
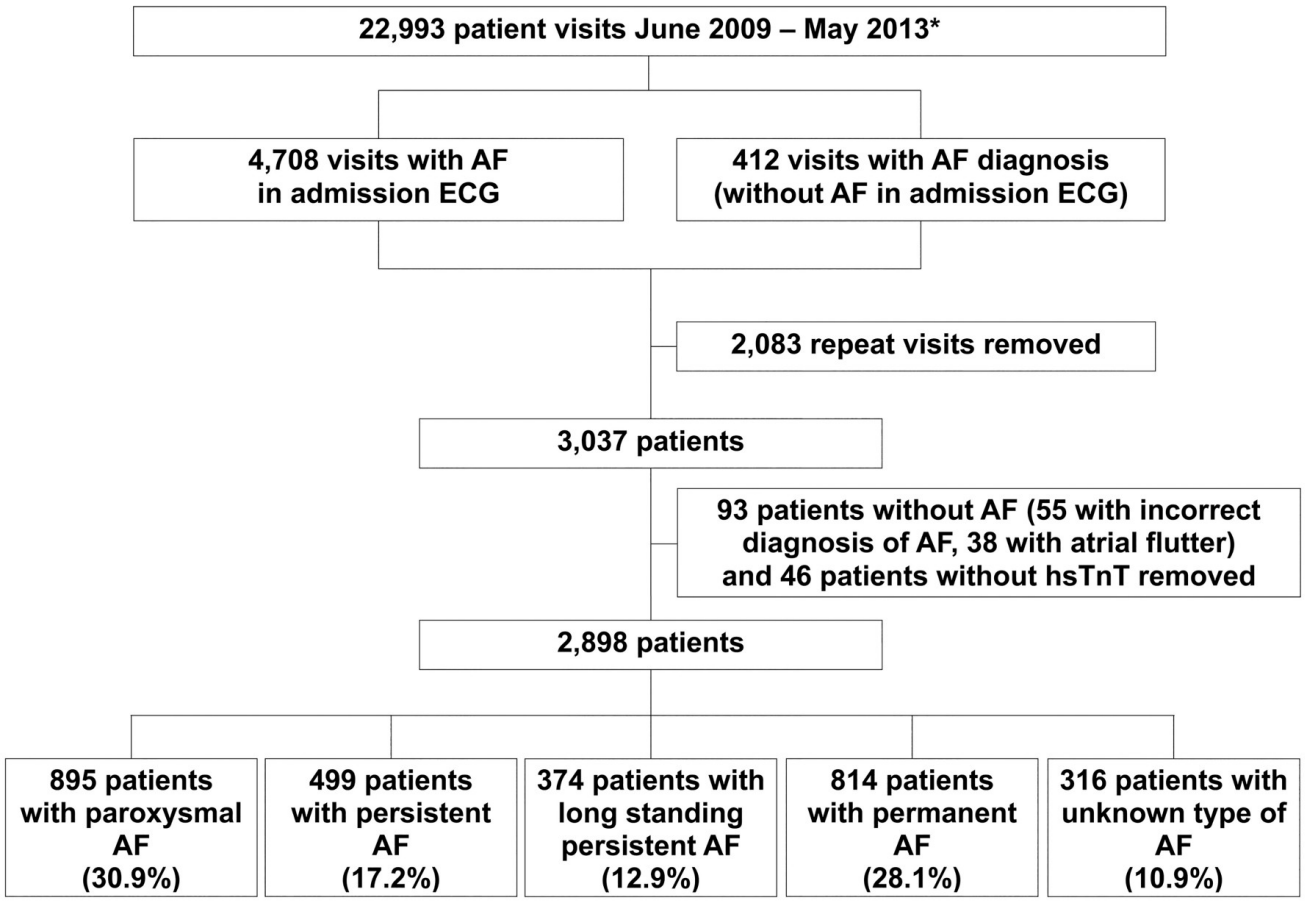
Diagram of patient inclusion. * Medical record software not in use from 11 November 2011 to 9 December 2011 AF denotes atrial fibrillation, hsTnT high-sensitivity troponin T. (Reproduced with permission from Oxford University Press [18]).

All patients admitted to the emergency department of the Department of Cardiology of the Heidelberg University Hospital from June 2009 to May 2013 with AF in the admission electrocardiogram (ECG) or history of AF were included. Patients presenting from 11th November 2011 to 9th December 2011 could not be included, since electronic medical record software was unavailable for this period. There were 22 993 visits to our emergency department, where hsTnT is routinely measured upon admission in all patients. There were 5120 visits of patients with AF (4708 patients had AF in the admission ECG and 412 patients had no AF in the admission ECG but a history of AF). Of these, 2083 were removed due to repeat visits, 93 were removed since detailed evaluation showed incorrect diagnosis of AF, and 46 patients were removed, since hsTnT upon admission was unavailable. The final study population consisted of 2898 patients (Fig 1). This retrospective observational study had no influence on patient treatment. All treatments were provided at the discretion of the treating physicians.

This study was approved by the Ethics Committee of the Medical Faculty of Heidelberg, requiring no informed consent for this retrospective analysis of patients from our own institution, using data from routine care. This study was conducted according to ethical principles stated in the Declaration of Helsinki (2008). Patient identifiable data was pseudonymized to ensure data confidentiality and was not passed on to third parties. This study is registered at ClinicalTrials.gov (NCT02542189).

Follow-up was performed by review of in- and outpatient visits to our clinic via hospital information system and review of results of routine medical aftercare, which is performed via patient phone calls, postal queries with standardized questionnaires, contact with primary care physicians and letters to registration offices. Patients without follow-up on ischemic stroke were not included in the survival analysis regarding this endpoint. All reported events were adjudicated by a physician after a follow-up of at least 1 month up to 60 months. Patients with hemorrhagic stroke were excluded.

HsTnT was measured on Cobas E411 (Roche Diagnostics Ltd., Rotkreuz, Switzerland). Limit of blank (3 ng/L) and limit of detection (5 ng/L) were determined in accordance with CLSI guideline EP17-A. Details on assay performance and analytical characteristics have previously been reported [19]. Normal reference values were established in a multicenter reference study and the 99th percentile value was determined at 14 ng/L [19]. Assay performance was not re-assessed in our central laboratory. None of the lots used in our central laboratory were affected by a lot-to-lot variation issue that has been reported for hsTnT [20]. The diagnosis of acute myocardial infarction was based on the third universal definition of myocardial infarction, requiring a rise and/or fall of hsTnT with at least one value above the 99th percentile (14 ng/L) [21].

Baseline characteristics are reported for the overall group as well as for two subgroups based on hsTnT levels: hsTnT at or below the 99th percentile and hsTnT above the 99th percentile (elevated hsTnT).

### CHA_2_DS_2_-VASc score

CHA_2_DS_2_-VASc score was calculated for all patients by adding two points for age ≥ 75 years and prior stroke or transient ischemic attack, respectively; as well as one point for congestive heart failure (left ventricular ejection fraction < 45% or main admission diagnosis of heart failure), hypertension, age between 65–74 years, diabetes mellitus, prior myocardial infarction or peripheral artery disease, and female gender, respectively [5].

### Statistical analysis

Data are presented as means (standard deviations), medians (25th, 75th percentiles), Kaplan-Meier estimates, counts or percentages. Continuous variables were tested for normal distribution with the Kolmogorov-Smirnov test. Groups were compared using chi-squared test or Fisher’s exact test for categorical variables, and unpaired Student’s t-test or Wilcoxon rank-sum test for continuous variables. Kaplan-Meier analysis was performed and groups were compared with the log-rank test. A multivariate Cox proportional hazards regression was performed to determine predictors for stroke. The proportional hazards assumption was tested using the Grambsch and Therneau method [22]. Time-dependent receiver-operating-characteristic (ROC) curves from censored survival data using the Kaplan-Meier method were estimated and the area under the ROC curves (AUC) was calculated [23]. The 95% confidence interval (CI) of AUC was calculated according to Hanley and McNeil [24]. To assess the added value of hsTnT regarding stroke prediction, measures for evaluating its incremental value, integrated discrimination improvement (IDI) and continuous net reclassification improvement (NRI) were calculated using censored time-to-event data [25]. A two tailed P-value of <0.05 was considered to indicate statistical significance. All statistical analyses were performed using R 3.3.3 (The R Foundation for Statistical Computing, Vienna, Austria).

## Results

### Baseline characteristics

Baseline characteristics are reported for the overall group as well as for both subgroups based on hsTnT level: hsTnT at or below the 99th percentile and hsTnT above the 99th percentile (elevated hsTnT) (Table 1). The groups differed significantly with respect to the baseline variables. The majority of patients were male (58.8%). Median age increased with higher troponin-values with a median of 78 years in patients with elevated hsTnT. Cardiovascular risk factors were more prevalent in patients with hsTnT above the 99th percentile.

**Table 1:** Baseline characteristics.

	All Patients (n = 2898)	hsTnT ≤ 14 ng/L (n = 1267)	hsTnT > 14 ng/L (n = 1631)	P-Value
Age (years)	74 [66–81]	69 [59–75]	78 [71–84]	< 0.001
Male sex	1704/2898 (58.8)	699/1267 (55.2)	1005/1631 (61.6)	<0.001
Diabetes mellitus	785/2887 (27.2)	222/1263 (17.6)	563/1624 (34.7)	< 0.001
Arterial hypertension	2368/2884 (82.1)	952/1263 (75.4)	1416/1621 (87.4)	< 0.001
Prior stroke or transient ischemic attack	298/2887 (10.3)	89/1265 (7.0)	209/1622 (12.9)	< 0.001
Peripheral artery disease	237/2886 (8.2)	49/1264 (3.9)	188/1622 (11.6)	< 0.001
Coronary artery disease	1322/2880 (45.9)	420/1262 (33.3)	902/1618 (55.7)	< 0.001
Prior myocardial infarction	501/2885 (17.4)	116/1264 (9.2)	385/1621 (23.8)	< 0.001
Prior percutaneous coronary intervention	604/2878 (21.0)	189/1264 (15.0)	415/1615 (25.7)	< 0.001
Prior coronary artery bypass surgery	277/2886 (9.6)	60/1264 (4.7)	217/1622 (13.4)	< 0.001
Reduced ejection fraction (moderately or severely, < 45%)	601/1787 (33.6)	115/669 (17.2)	486/1118 (43.5)	< 0.001
Valvular atrial fibrillation	186/2475 (7.5)	38/1102 (3.4)	148/1373 (10.8)	< 0.001
Atrial fibrillation in admission electrocardiogram (ECG)	2628/2891 (90.9)	1110/1265 (87.7)	1518/1626 (93.4)	< 0.001
Estimated glomerular filtration rate (eGFR)* (mL/min/1.73 m^2^)	71 [51–88]	82 [67–97]	59 [41–79]	< 0.001
Severely impaired renal function (eGFR < 30 mL/min/1.73 m^2^)	208/2888 (7.2)	8/1260 (0.6)	200/1628 (12.3)	< 0.001
Liver cirrhosis	25/2884 (0.9)	9/1265 (0.7)	16/1619 (1.0)	0.553
Aspartate transaminase (AST) (IU/L)	27 [20–37]	25 [20–33]	28 [21–42]	< 0.001
Aspartate transaminase (AST) > 2 x upper limit of normal (ULN)	138/2863 (4.8)	32/1254 (2.6)	106/1609 (6.6)	< 0.001
Prior major bleeding	146/2875 (5.1)	36/1265 (2.8)	110/1610 (6.8)	< 0.001

Data are median [25th, 75th percentiles] or number of patients (%)

* By Modification of Diet in Renal Disease (MDRD) equation

The majority of patients had AF in the admission ECG (90.9%) and AF was the most frequent main admission diagnosis (42.7%). The frequencies of other admission diagnoses are listed in Table 2. At discharge, 6.7% of all patients (3.6% of patients with hsTnT at or below the 99th percentile and 9.3% of patients with elevated hsTnT, p < 0.001) were treated with triple therapy (oral anticoagulation, aspirin and a P2Y_12_ inhibitor).

**Table 2:** Main admission diagnoses.

	All Patients (n = 2898)	hsTnT ≤ 14 ng/L (n = 1267)	hsTnT > 14 ng/L (n = 1631)	P-Value
Atrial fibrillation	1236/2898 (42.7)	819/1267 (64.6)	417/1631 (25.6)	< 0.001
Heart failure	307/2898 (10.6)	60/1267 (4.7)	247/1631 (15.1)	< 0.001
Hypertensive emergency	83/2898 (2.9)	45/1267 (3.6)	38/1631 (2.3)	0.065
Infection	182/2898 (6.3)	32/1267 (2.5)	150/1631 (9.2)	< 0.001
Unstable angina	146/2898 (5.0)	58/1267 (4.6)	88/1631 (5.4)	0.361
Non-ST-segment elevation myocardial infarction	203/2898 (7.0)	6/1267 (0.5)	197/1631 (12.1)	< 0.001
ST-segment elevation myocardial infarction	35/2898 (1.2)	3/1267 (0.2)	32/1631 (2.0)	< 0.001
Other diagnoses	706/2898 (24.4)	244/1267 (19.3)	462/1631 (28.3)	< 0.001

Data are number of patients (%)

**Fig 2:**
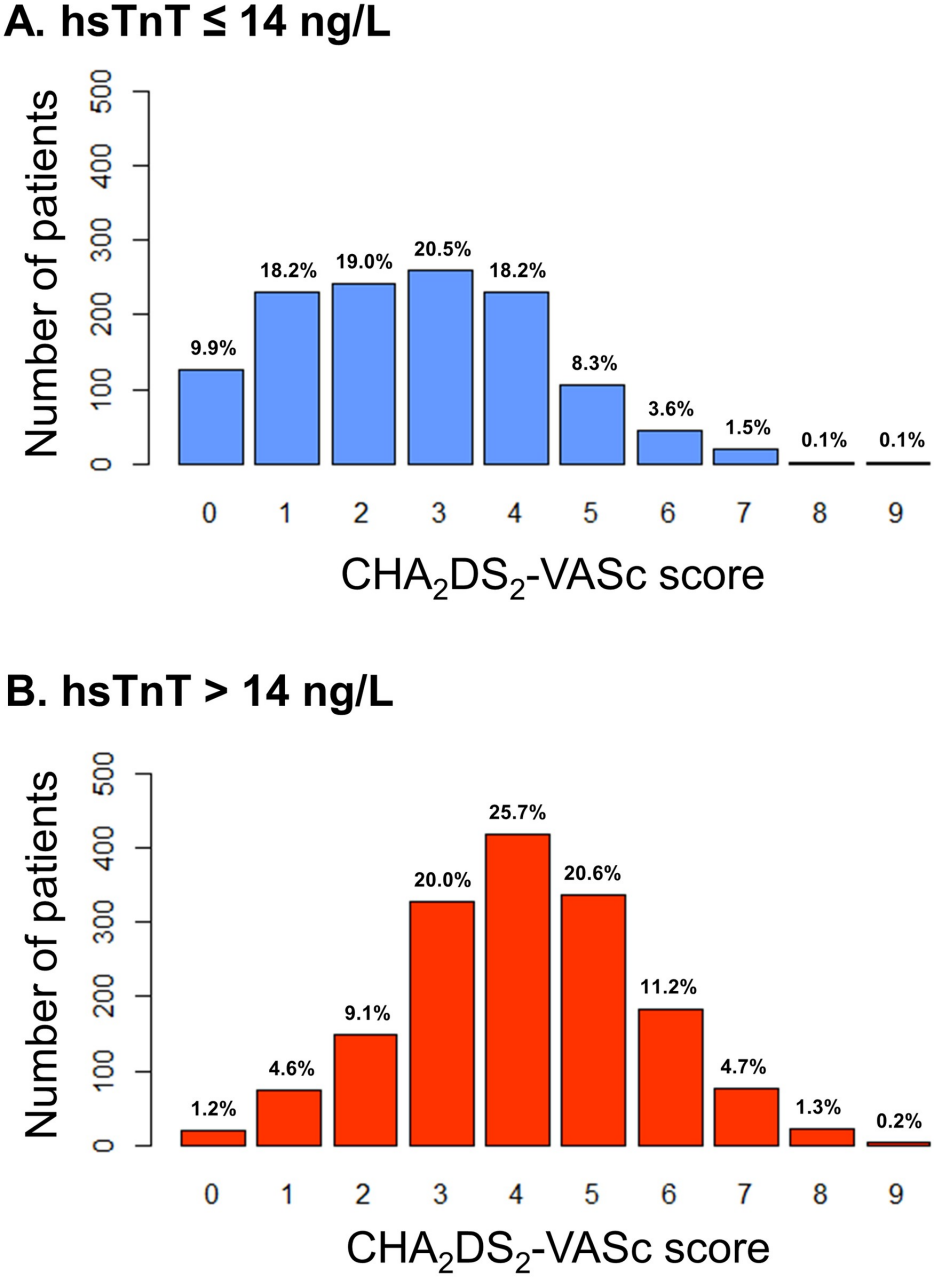
Distribution of CHA_2_DS_2_-VASc score in patients with atrial fibrillation for the subgroups of hsTnT under the 99th percentile (A. hsTnT ≤ 14 ng/L), and elevated hsTnT (B. hsTnT > 14 ng/L). hsTnT = high-sensitivity troponin T.

CHA_2_DS_2_-VASc score was available in 99.0% of patients. The majority of patients included in this study had a CHA_2_DS_2_-VASc score of 2 or higher (83.4%). A total of 71.3% of patients with hsTnT at or below the 99th percentile and 92.8% of patients with hsTnT above the 99th percentile had a CHA_2_DS_2_-VASc score of 2 or higher (Fig 2).

Median follow-up duration for stroke was 710.0 days [25th– 75th percentiles: 289.5–1061.5 days]. Follow-up information on vital status and stroke could be obtained in 94.3% and 76.7%, respectively. On admission, median hsTnT was 17.0 ng/L (25th percentile 9.0 ng/L, and 75th percentile 37.0 ng/L) and mean hsTnT was 67.8 ng/L.

### Prognostic value of high-sensitivity cardiac troponin T

**Fig 3:**
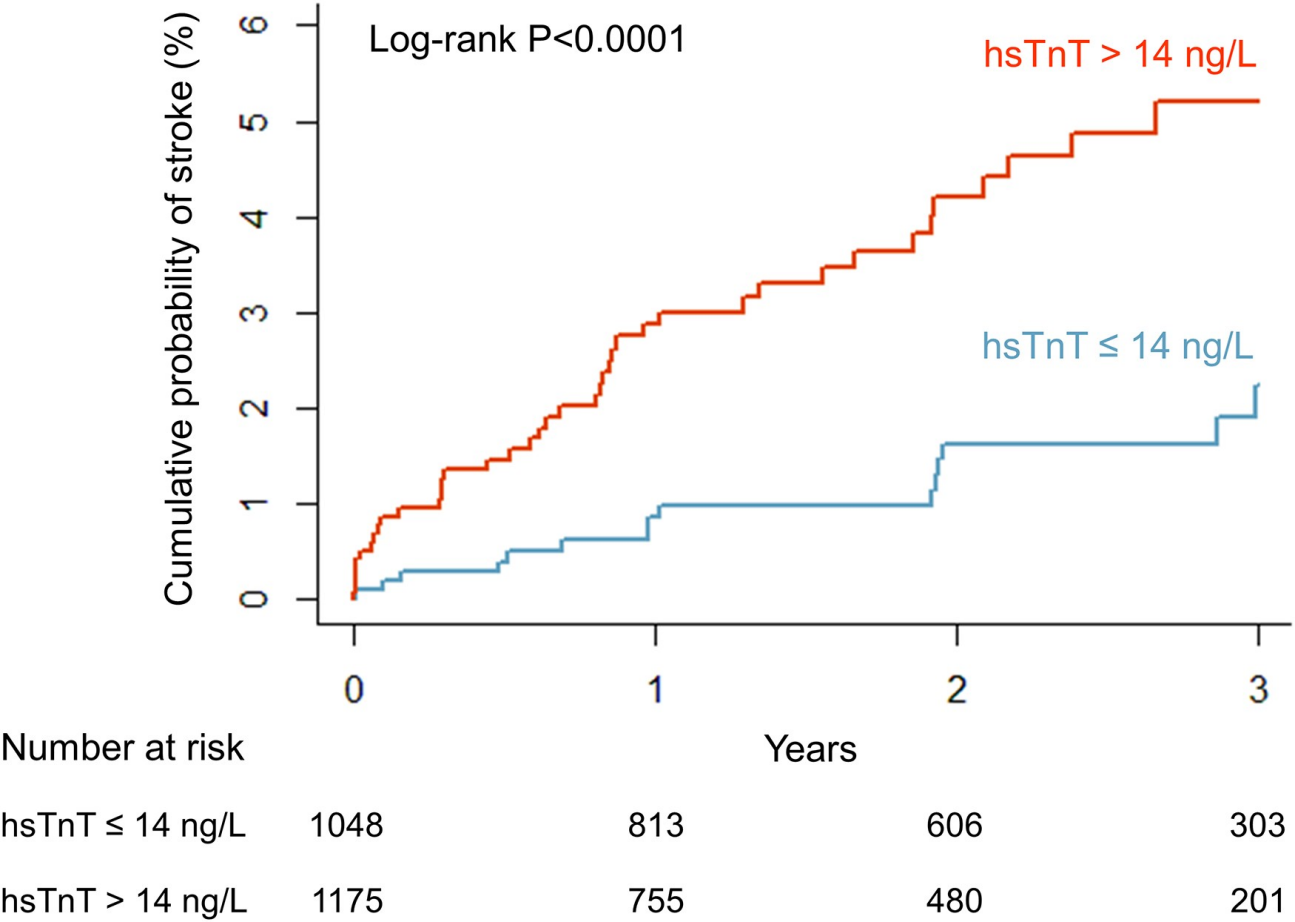
Kaplan-Meier curve for stroke in patients with atrial fibrillation: hsTnT under the 99th percentile (hsTnT ≤ 14 ng/L, blue), and elevated hsTnT (hsTnT > 14 ng/L, red). hsTnT = high-sensitivity troponin T.

In total, there were 60 cases of stroke. In the subgroup of patients with hsTnT at or below the 99th percentile there were 16 cases of stroke and in patients with elevated hsTnT there were 44 cases (Kaplan–Meier estimates for stroke at 1 year 0.9%, and 2.9%, respectively). Kaplan–Meier curves are shown in Fig 3. A log-rank test for the two hsTnT-subgroups was significant (P<0.001).

A multivariate Cox proportional hazards model was used to determine predictors for stroke in patients with AF. HsTnT, as well as all variables of the CHA_2_DS_2_-VASc score were included. Among these, elevated hsTnT levels, higher age, and prior stroke or transient ischemic attack were significantly associated with an increased risk of stroke in patients with AF. Adjusted hazard ratio for elevated hsTnT was 2.35 [95% CI: 1.26–4.36] (P = 0.007) (Table 3).

**Table 3:** Results of Cox proportional-hazards regression for predictors of stroke in patients with atrial fibrillation.

	Adjusted Hazard Ratio [95% Confidence Interval]	P-Value
Elevated high-sensitivity cardiac troponin T (> 14 ng/L)	2.35 [1.26–4.36]	0.007
Congestive heart failure*	1.04 [0.58–1.85]	0.905
Hypertension	0.97 [0.43–2.19]	0.945
Age ≥ 75 years (versus < 65 years)	3.35 [1.12–10.02]	0.031
Age 65–74 years (versus < 65 years)	3.57 [1.21–10.52]	0.021
Diabetes mellitus	1.47 [0.86–2.51]	0.161
Prior stroke or transient ischemic attack	2.06 [1.06–4.00]	0.032
Prior myocardial infarction or peripheral artery disease	0.82 [0.44–1.55]	0.544
Sex (female)	1.19 [0.69–2.05]	0.529

* Left ventricular ejection fraction < 45% or main admission diagnosis of heart failure

The inclusion of hsTnT in the multivariable model for stroke prediction, which included all variables of CHA_2_DS_2_-VASc score, was associated with a significant improvement of its performance. The inclusion of elevated hsTnT resulted in an IDI 0.004 [95% CI: 0.001–0.012] (P = 0.027) and continuous NRI 0.244 [95% CI: 0.061–0.385] (P = 0.013) for 1-year stroke risk.

**Fig 4:**
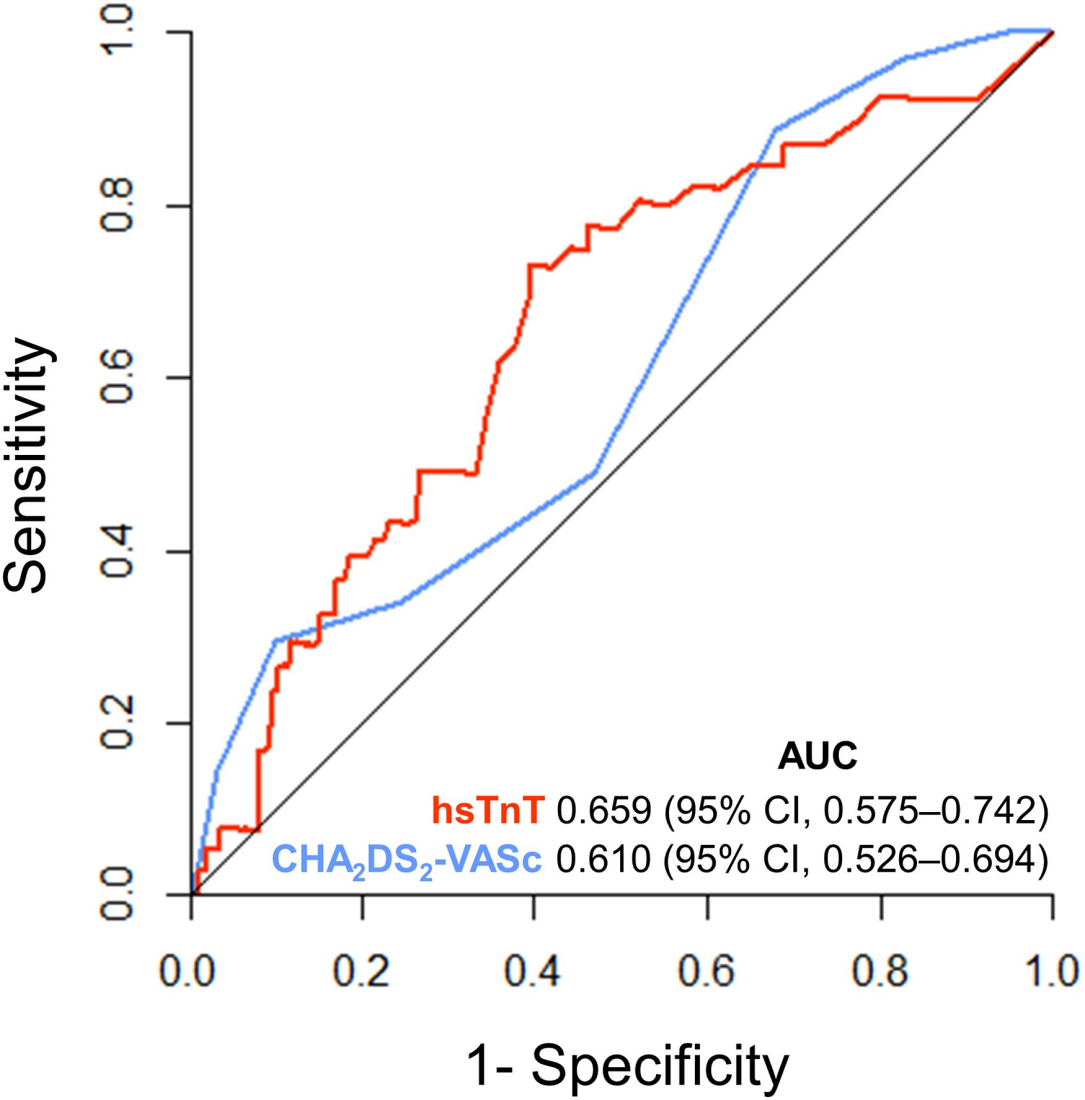
Receiver operating characteristic (ROC) curves for prediction of stroke by hsTnT (red) and CHA_2_DS_2_-VASc score (blue) in patients with atrial fibrillation (P = 0.250). AUC = area under the curve, hsTnT = high-sensitivity troponin T, 95% CI = 95% confidence interval.

The diagnostic performance of hsTnT regarding stroke at 1 year was reflected by the AUC of 0.659 [95% CI: 0.575–0.742], as compared to 0.610 [95% CI: 0.526–0.694] for the CHA_2_DS_2_-VASc score (P = 0.250) (Fig 4). For the combination of hsTnT and CHA_2_DS_2_-VASc score, AUC was 0.580 [95% CI: 0.560–0.601].

Calculating time-dependent ROC curves for censored time-to-event data, the maximum value of the Youden’s Index was determined at a cut-off value of hsTnT 19.23 ng/L (sensitivity 73.1%, specificity 60.5%, positive predictive value 3.4% and negative predictive value 99.1% at 1 year).

Elevated hsTnT levels were also significantly associated with an increased risk of stroke after exclusion of patients with MI (NSTEMI and STEMI) as main admission diagnosis (2660 patients after exclusion of MI). In this subgroup the adjusted HR for elevated hsTnT (> 14 ng/ L) was 2.06 [95% CI: 1.07–3.95] (P = 0.030).

In a ROC-curve analysis regarding stroke after exclusion of MI patients, hsTnT had an AUC of 0.643 [95% CI: 0.559–0.727], the CHA_2_DS_2_-VASc score an AUC of 0.639 [95% CI: 0.555–0.723], and the combination of hsTnT and CHA_2_DS_2_-VASc score an AUC of 0.613 [95% CI: 0.592–0.635], showing results similar to those of the entire population. After exclusion of patients with MI, maximum value of the Youden’s Index was calculated at the same cut-off value of hsTnT 19.23 ng/L (sensitivity 66.5%, specificity 64.7%, positive predictive value 3.0% and negative predictive value 99.0% at 1 year).

**Fig 5:**
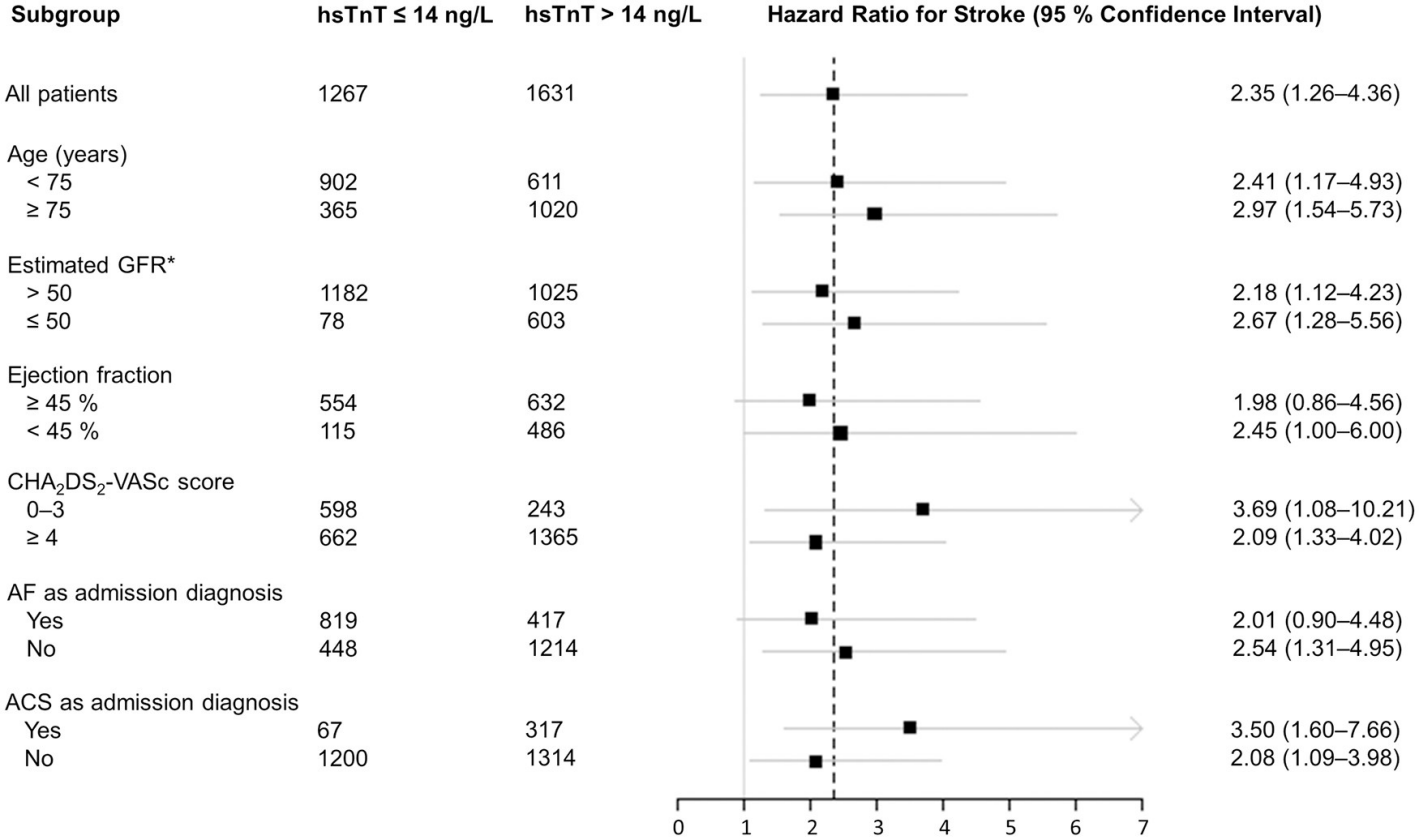
Stroke risk in patients with atrial fibrillation and subgroups for elevated hsTnT (hsTnT > 14 ng/L) compared to hsTnT under the 99th percentile (hsTnT ≤ 14 ng/L). ACS = acute coronary syndrome, AF = atrial fibrillation, GFR = glomerular filtration rate, hsTnT = high-sensitivity troponin T. * By Modification of Diet in Renal Disease (MDRD) equation (mL/min/1.73 m^2^).

An overall consistency of the hazard was observed across relevant subgroups of patients. There were no significant interactions regarding important potential confounders, such as age, renal function, ejection fraction, CHA_2_DS_2_-VASc score, AF as main admission diagnosis or ACS at admission (Fig 5).

In the subgroup of female patients with CHA_2_DS_2_-VASc score 0, 1 and 2 and male patients with CHA_2_DS_2_-VASc score 0 and 1, there were only 2 stroke events–one of them in the group of elevated hsTnT, the other in the group of hsTnT at or below 99th percentile (S1 Fig).

## Discussion

The present study of consecutive patients with AF presenting to an emergency department shows that elevated hsTnT levels are significantly associated with higher risk of stroke. In the model including all CHA_2_DS_2_-VASc score variables, hsTnT remains independently associated higher stroke risk and provides added prognostic information independent of other major risk factors and clinical characteristics. The inclusion of hsTnT is associated with a significant improvement of the discriminatory power of a model consisting of the CHA_2_DS_2_-VASc score variables.

The underlying mechanisms of why elevated troponin levels help identify patients at risk for stroke are not fully understood, but likely to be numerous and not only related to acute myocardial ischemia. Possible explanations for troponin elevations in patients with AF include myocardial injury due to high ventricular rates in tachyarrhythmia, microvascular blood flow alterations and oxidative stress [26]. An association of elevated troponin levels with extensive atherosclerosis, carotid lesions, and increased left ventricular filling pressures has also been reported, which may mediate a higher risk for stroke.

The prognostic value of biomarkers in patients with AF regarding stroke risk prediction has been addressed in several previous studies. Secondary analyses of RCTs comparing the efficacy and safety of novel oral anticoagulants (NOACs) with warfarin provided evidence for association of elevated cardiac troponin levels with adverse outcomes in patients with AF, including increased stroke risk. In a substudy of ARISTOTLE trial, high-sensitivity troponin-I was significantly associated with stroke or systemic embolism with an adjusted HR of 1.98 [95% CI: 1.42–2.78] [27]. In another analysis of the ARISTOTLE trial, annual rates of stroke or systemic embolism were significantly higher in the highest high-sensitivity troponin T quartile compared with the lowest quartile (adjusted HR 1.94 [95% CI: 1.35–2.78]) [14].

Recently, biomarker-based risk scores for patients with AF have been introduced. The Age, Biomarkers, Clinical history (ABC) score was derived from a cohort of 14 701 patients from the ARISTOTLE trial [15]. In a subanalysis of the Effective Anticoagulation with Factor Xa Next Generation in Atrial Fibrillation–Thrombolysis in Myocardial Infarction 48 (ENGAGE AF-TIMI 48) trial, a multimarker risk score including cardiac troponin I, N-terminal pro-B-type natriuretic peptide, and D-dimer levels was developed [16]. When added to the CHA_2_DS_2_-VASc score, the biomarker score based on the ENGAGE AF-TIMI 48 trial significantly enhanced prognostic accuracy by improving the C statistic from 0.586 [95% CI: 0.565–0.607] to 0.708 [95% CI: 0.688–0.728] (P < 0.001) and reclassification with a net reclassification improvement of 59.4% (P < 0.001).

The findings of the present study are novel and add information to available data for several reasons. First, data was collected from unselected patients presenting to an emergency department, including patients with clinical characteristics, many of which had been excluded in previous randomized controlled trials. Patients with valvular AF, severely impaired renal function, liver disease, previous major bleeding and triple therapy (simultaneous treatment with oral anticoagulation, aspirin and a P2Y_12_ inhibitor) were all included, therefore better reflecting the real world setting.

Second, we were able to investigate the prognostic role of hsTnT across a wide spectrum of cardiac comorbidities that may contribute to hsTnT elevation and could also explain the prognostic impact of hsTnT in AF. HsTnT was significantly associated with higher stroke risk independently of important risk factors and clinical characteristics. Furthermore, a subgroup analysis of the present study showed that the prognostic impact of elevated hsTnT in AF is consistent throughout relevant subgroups–no significant interactions were observed regarding age, renal function, ejection fraction, CHA_2_DS_2_-VASc score, AF as main admission diagnosis or ACS at admission.

Third, the present study addresses the role of hsTnT for stroke prediction in relation to established clinical risk factors and especially the guideline recommended CHA_2_DS_2_-VASc risk score in patients with AF. Our results confirm that the CHA_2_DS_2_-VASc risk score has a modest performance regarding stroke prediction with an AUC of 0.610 [95% CI: 0.526–0.694], compared to hsTnT with an AUC of 0.659 [95% CI: 0.575–0.742], the difference was not statistically significant (P = 0.250). Furthermore, elevated hsTnT was not only independently associated with stroke risk in patients with AF, but its inclusion to the multivariable model for stroke risk prediction consisting of all variables of the CHA_2_DS_2_-VASc score was associated with a significant improvement of its discriminatory power. By providing prognostic information independent of other clinical variables, hsTnT may be beneficial for stroke risk assessment in patients with AF. Further studies should evaluate hsTnT as a variable in risk scores for stroke risk prediction.

There are some limitations to the present study. Since only hsTnT was evaluated, conclusions on the role of conventional cardiac troponin T, conventional cardiac troponin I or high-sensitivity cardiac troponin I assays are not possible. The reported events were adjudicated by a single physician with the possible consequence of an observer bias. Bias from potentially missed events in patients without follow-up for stroke cannot be excluded. This is due to the fact that municipal registration offices had to be contacted in some cases, where only a follow-up for mortality could be obtained.

## Conclusions

The present study shows that elevated hsTnT is independently associated with higher rates of stroke in patients presenting to an emergency department with AF. Furthermore, hsTnT provides prognostic information independent of CHA_2_DS_2_-VASc risk score variables. In the light of these findings, hsTnT could serve as a valuable prognostic biomarker and its measurement should be considered for stroke risk prediction as compared to only using clinical variables. Further studies are warranted to evaluate the value of cardiac biomarkers as part of combined risk-scores or algorithms for stroke risk prediction, incorporating both clinical and biochemical variables.

## Supporting information

S1 Fig Kaplan-Meier curve for stroke in patients with atrial fibrillation, subgroup of patients with CHA_2_DS_2_-VASc score ≤ 2 (female) and CHA2DS2-VASc score ≤ 1 (male): hsTnT at or below the 99th percentile (hsTnT ≤ 14 ng/L, blue), and elevated hsTnT (hsTnT > 14 ng/L, red). hsTnT = high-sensitivity troponin T.
(TIF)
