# Supplementary figures and images for: High-sensitivity cardiac troponin T as an independent predictor of stroke in patients admitted to an emergency department with atrial fibrillation
Source: PLoS One. 2019 Feb 12;14(2):e0212278. doi: 10.1371/journal.pone.0212278 (PMC6372209; doi:10.1371/journal.pone.0212278)

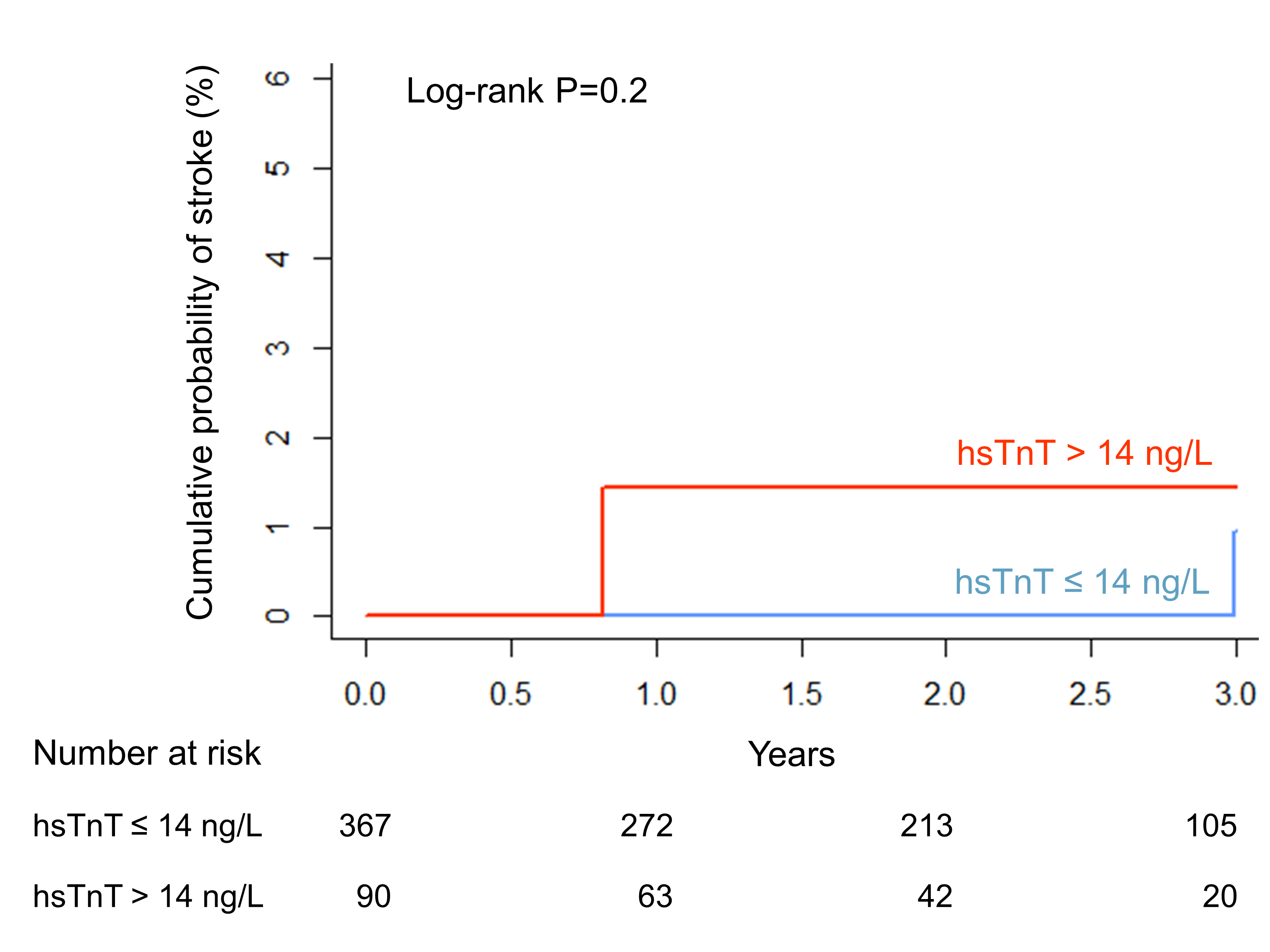

Supplement: S1 Fig — hsTnT = high-sensitivity troponin T. (TIF) [file pone.0212278.s001.tif]
